# Formación de médicos generales en América Latina: un reto para la salud universal

**DOI:** 10.26633/RPSP.2019.83

**Published:** 2019-10-15

**Authors:** Germán Fajardo Dolci, Javier Santacruz Varela, Iván F. Contrera Toro, Marcelo A. Yorio Nieto, Luis A. Pichs García, Guido W. Zambrana Ávila, Freddy A. Meynard Mejía, Eleazar Lara Padilla

**Affiliations:** 1 Facultad de Medicina, Universidad Nacional Autónoma de México Facultad de Medicina, Universidad Nacional Autónoma de México Ciudad de México México Facultad de Medicina, Universidad Nacional Autónoma de México, Ciudad de México, México.; 2 Departamento de Proyectos Especiales, Facultad de Medicina de la UNAM, México Departamento de Proyectos Especiales, Facultad de Medicina de la UNAM, México Ciudad de México México Departamento de Proyectos Especiales, Facultad de Medicina de la UNAM, México, Ciudad de México, México.; 3 Faculdade de Ciencias Médicas, Universidad de Campinas Faculdade de Ciencias Médicas, Universidad de Campinas Campinas Brasil Faculdade de Ciencias Médicas, Universidad de Campinas, Campinas, Brasil.; 4 Facultad de Ciencias Médicas, Universidad Nacional de Córdoba Facultad de Ciencias Médicas, Universidad Nacional de Córdoba Córdoba Argentina Facultad de Ciencias Médicas, Universidad Nacional de Córdoba, Córdoba, Argentina.; 5 Universidad de Ciencias Médicas de la Habana Universidad de Ciencias Médicas de la Habana La Habana Cuba Universidad de Ciencias Médicas de la Habana, La Habana, Cuba.; 6 Facultad de Medicina, Universidad Mayor de San Andrés Facultad de Medicina, Universidad Mayor de San Andrés La Paz Bolivia Facultad de Medicina, Universidad Mayor de San Andrés, La Paz, Bolivia.; 7 Facultad de Ciencias Médicas, Universidad Nacional Autónoma de Nicaragua Facultad de Ciencias Médicas, Universidad Nacional Autónoma de Nicaragua Managua Nicaragua Facultad de Ciencias Médicas, Universidad Nacional Autónoma de Nicaragua, Managua, Nicaragua.; 8 Escuela Superior de Medicina, Instituto Politécnico Nacional Escuela Superior de Medicina, Instituto Politécnico Nacional Ciudad de México México Escuela Superior de Medicina, Instituto Politécnico Nacional, Ciudad de México, México.

**Keywords:** Recursos humanos, educación médica, cobertura universal de salud, América Latina, Workforce, medical education, universal health coverage, Latin America, Recursos humanos, educação médica, cobertura universal de saúde, América Latina

## Abstract

**Objetivo.:**

Conocer la opinión de las escuelas de medicina sobre la existencia de políticas públicas y la suficiencia de financiamiento público y mecanismos regulatorios para la formación médica de pregrado e identificar áreas que permitan mejorar la disponibilidad de médicos generales en la Región de las Américas.

**Métodos.:**

Estudio transversal, descriptivo realizado con 105 escuelas de medicina, 51 públicas y 54 privadas, de 17 países. Se utilizó un cuestionario con una escala tipo Likert para explorar tres dimensiones (contextos político, económico y regulatorio) integradas por 4, 2 y 4 variables cada una, respectivamente, y validado con el método Delphi. Se estimaron frecuencias de respuestas a las preguntas del cuestionario. Se realizó un análisis de frecuencias, y para identificar diferencias entre escuelas públicas y privadas se efectuó un análisis bivariante aplicando la prueba de Chi cuadrado para comparar porcentajes.

**Resultados.:**

El contexto político fue favorable para 64% de las escuelas, el contexto económico, para 37% y la regulación, para 23%. Sólo hubo diferencias significativas entre escuelas públicas y privadas en la variable recursos financieros que ellas administran.

**Conclusiones.:**

Es necesario fortalecer las políticas públicas, la inversión pública y la regulación de la educación médica, para mejorar la formación y disponibilidad de médicos generales en los países de la Región.

## Abstract (en)

**Objective.:**

To gather opinions from medical schools regarding the existence of public policies on the health workforce (human resources for health) and whether sufficient public financing and regulatory mechanisms are in place for undergraduate medical education; and to identify areas of opportunity to improve the availability of general practitioners in the Region of the Americas.

**Methods.:**

Cross-sectional, descriptive study conducted with 105 medical schools (51 public and 54 private) in 17 countries. A questionnaire with a Likert scale was used to explore three dimensions (political, economic, and regulatory contexts) composed of 4, 2, and 4 variables each, respectively, and validated with the Delphi method. Frequencies of responses to the questions were estimated. A frequency analysis was performed, as well as a bivariate analysis to identify differences between public and private schools, applying the Chi-square test to compare percentages.

**Results.:**

The political context was considered favorable by 64% of the schools; the economic context, by 37%; and the regulatory context, by 23%. The only significant differences between public and private schools were in the financial resources they administer.

**Conclusions.:**

It is necessary to strengthen public policies, public investment, and the regulation of medical education in order to improve the education and availability of general practitioners in the countries of the Region.

## RESUMO

**Objetivo.:**

Conhecer a opinião das faculdades de medicina sobre o volume de políticas públicas e financiamento público e mecanismos reguladores para graduação médica e identificar áreas que possibilitem aumentar o número de clínicos gerais na Região das Américas.

**Métodos.:**

Estudo transversal descritivo realizado com 105 faculdades de medicina (51 públicas e 54 particulares) em 17 países. Um questionário com uma escala tipo Likert foi usado para explorar três dimensões (contexto político, contexto econômico e regulamentação), contendo 4, 2 e 4 variáveis cada, e foi validado com o método Delphi. As frequências de respostas às perguntas do questionário foram calculadas e analisadas. A fim de identificar diferenças entre as faculdades públicas e particulares, uma análise bivariada com teste qui-quadrado foi realizada para comparar porcentagens.

**Resultados.:**

O contexto político foi considerado favorável por 64% das faculdades; o contexto econômico por 37%; e a regulamentação por 23%. Apenas foi observada diferença significativa entre as faculdades públicas e particulares na variável recursos financeiros geridos.

**Conclusões.:**

É necessário fortalecer as políticas públicas, o investimento público e a regulamentação da educação médica para melhorar a formação e aumentar o número de clínicos gerais nos países da Região.

La salud universal es indispensable para el desarrollo humano sostenible y uno de los factores para conseguirla es la adecuada disponibilidad y distribución de personal de salud en los países. En 2006, la Organización Mundial de la Salud (OMS) alertó sobre el déficit de 4,3 millones de trabajadores de salud, que en 2013 aumentó a siete millones y se prevé que llegará a 15 millones en 2030. Esta deficiencia se agudiza por su irregular distribución y concentración en las zonas urbanas (1-3).

La escasez de personal de salud es un factor que dificulta el logro de la salud universal y esta dificultad se ha analizado en los Foros Mundiales de Recursos Humanos para la Salud. En el primero de ellos celebrado en 2008, se aprobó la Declaración de Kampala, en la cual se insta a los gobiernos a atender el déficit de personal sanitario (4), en el segundo, de 2011, se recomendó mejorar el financiamiento para garantizar la formación de personal de salud (5), y en el tercero, de 2013, se aprobó la Declaración política de Recife sobre Recursos Humanos para la Salud, en la cual se destaca el papel central que desempeñan de los recursos humanos para lograr la salud universal (6).

Para atender este déficit y alcanzar las metas de salud de los Objetivos de Desarrollo Sostenible, las Naciones Unidas aprobaron en 2015 la Resolución 70/220 sobre Desarrollo de Recursos Humanos, que exhorta a los Estados Miembros a “formular estrategias a corto, mediano y largo plazo que mejoren de manera eficaz sus capacidades en materia de recursos humanos” (7). La OMS por su parte aprobó en 2016 la Estrategia Mundial de Recursos Humanos para la Salud: personal sanitario 2030, la cual insta a los Estados Miembros a subsanar las disparidades entre necesidades, demanda y oferta de personal de salud, así como a mejorar su distribución (8). Para reducir el déficit de 800 000 trabajadores de la salud en la Región de las Américas, la Organización Panamericana de la Salud (OPS) aprobó en 2018 el Plan de Acción sobre Recursos Humanos para el Acceso Universal a la Salud y la Cobertura Universal de Salud 2018-2023 *(9).*

Los médicos generales son una parte fundamental de los recursos humanos de salud y su formación está a cargo de las escuelas de medicina. Por ser un proceso de interés social, la educación médica durante el pregrado requiere políticas públicas educativas y económicas que le den direccionalidad y sostenibilidad financiera (10). Por otra parte, la educación médica se desarrolla en un contexto demográfico, epidemiológico y de servicios de salud cambiante. Para responder a dichos cambios, las escuelas de medicina modifican periódicamente las competencias profesionales de sus planes de estudio. Asimismo, y al igual que en la educación superior, en la educación médica interviene un conjunto de fuerzas culturales, sociales, políticas y económicas cuyos pesos se ponderan y a la postre determinan su intención y contenido (11)

A pesar de que cada vez se usan con mayor frecuencia métodos prospectivos para planificar la formación de médicos a mediano y largo plazo (12-14), no ha sido posible resolver su déficit y la disponibilidad de médicos sigue siendo menor a 1 por 1 000 habitantes en algunos países como Bolivia, Guatemala, Haití y Nicaragua (15). Esta escasez puede ser el reflejo de la falta de políticas públicas, de escaso presupuesto y de deficiente regulación para la formación de recursos humanos en salud, pero la información disponible no es suficiente para aseverarlo.

Ante esta situación, la Asociación Latinoamericana y del Caribe de Facultades y Escuelas de Medicina (ALAFEM) decidió realizar el presente estudio cuyo objetivo fue conocer la opinión de las escuelas de medicina sobre la existencia de políticas públicas, la suficiencia de financiamiento público, así como de mecanismos regulatorios para la formación médica durante el pregrado e identificar áreas de oportunidad que permitan mejorar la disponibilidad de médicos generales en la Región.

## MATERIALES Y MÉTODOS

Entre abril y septiembre de 2017, se realizó un estudio transversal, cuantitativo y descriptivo en 105 escuelas de medicina de América Latina que aceptaron participar, de un total de 617 (17%) que fueron invitadas a través de un mensaje por correo electrónico enviado a sus decanos, al que se adjuntó un resumen del protocolo del estudio aprobado (No. 111/2017) por el Comité de Ética de la División de Investigación de la Facultad de Medicina de la Universidad Nacional Autónoma de México. Las escuelas se identificaron en los directorios de las Asociaciones Nacionales de Escuelas de Medicina y del Directorio Mundial de Escuelas de Medicina de la World Federation for Medical Education. La distribución de escuelas participantes por región de ALAFEM, país y tipo de escuela se muestra en el cuadro 1.

**CUADRO 1.** Escuelas de medicina participantes públicas y privadas por región de ALAFEM y país, 2017

Región de ALAFEM	País	Públicas (No. total = 51)	Privadas (No. total = 54)	Participantes (No. total = 105)	Invitadas (No. total = 562)	Participación (%)
Andina	Bolivia	3	1	3	10	30,0
	Colombia	3	10	13	47	27,7
	Ecuador	3	6	9	22	40,9
	Perú	4	4	8	28	28,6
Brasil	Brasil	12	5	17	195	8,7
Caribe	Cuba	1	0	1	8	12,5
	Puerto Rico	1	0	1	4	25,0
	República Dominicana	0	2	2	12	16,7
Centroamérica	Costa Rica	1	1	2	7	28,6
	El Salvador	0	1	1	4	25,0
	Guatemala	0	1	1	4	25,0
	Nicaragua	2	1	3	7	42,9
Cono Sur	Argentina	4	3	7	34	20,6
	Chile	1	2	3	17	17,6
	Paraguay	1	1	2	6	33,3
	Uruguay	1	1	2	2	100,0
México	México	14	15	29	156	18,6

***Fuente***: Elaboración propia con los datos del estudio. ALAFEM: Asociación Latinoamericana y del Caribe de Facultades y Escuelas de Medicina.

Un grupo de 12 docentes a tiempo completo de seis escuelas propuestas por los Vocales del Consejo Directivo de la ALAFEM (una por cada una de sus regiones: México, Centroamérica, Zona Andina, Caribe, Brasil y Cono Sur) seleccionó diez variables relacionadas con tres dimensiones: el contexto político (existencia de políticas públicas para formar personal de salud; cuatro variables), el contexto económico (asignación de fondos públicos para la formación médica; dos variables), y el contexto regulatorio (existencia de normas y reglamentos para regular la formación de médicos generales; cuatro variables).

Asimismo, se diseñó un cuestionario con respuestas en una escala Likert, que se validó con el Método Delphi en el cual participaron 18 docentes de medicina de las mismas escuelas (su confiabilidad, estimada con la alfa de Cronbach fue 0,895). El cuestionario se colocó en una plataforma informática y al mismo respondió por consenso un grupo de 3 a 5 docentes de medicina de cada escuela seleccionados por el decano (370 docentes de las 105 escuelas).

Se realizó análisis descriptivo en el cual se calculó, para cada variable de las tres dimensiones, el porcentaje de respuestas a cada una de las siguientes cuatro categorías de respuesta: “total o parcialmente de acuerdo” (que se consideraron favorables) y “parcial o totalmente en desacuerdo” (que se consideraron desfavorables). Con la media de respuestas favorables de cada variable se obtuvo un resultado global para cada dimensión. Los porcentajes de respuestas entre escuelas públicas y privadas se compararon con la prueba de Chi cuadrado utilizando un nivel de significación estadística de 0,05. Los datos se registraron en hojas Excel y se analizaron con el programa SPSS.

## RESULTADOS

A continuación se presentan los resultados de todas las variables agrupadas según la dimensión a la cual pertenecen.

### Primera dimensión. Contexto político de la educación médica

Primera variable. Existencia de una política nacional de recursos humanos para la salud. El 77,1% de las escuelas consideró que existe esta política nacional y de ellas, 32,6% estuvo totalmente de acuerdo y 44,5% sólo de acuerdo.

Segunda variable. Utilidad de la política nacional de recursos humanos en salud para planificar la formación de médicos. El 46,8% de las escuelas consideró que la política nacional de recursos humanos para la salud es útil para guiar la planificación de médicos y de estas, 8,3% estuvo totalmente de acuerdo y 38,5% sólo de acuerdo. El 42,8% restante estuvo en desacuerdo y de estas, 10,4% en total desacuerdo.

Tercer**a** variable. Definición en la política nacional de salud del tipo de médicos que se debe formar. El 68,0% de las escuelas opinó favorablemente y de estas, 22% estuvo totalmente de acuerdo y 46% sólo de acuerdo. Del 32% que no opinó favorablemente, 7,0% estuvo en total desacuerdo.

**FIGURA 1.**
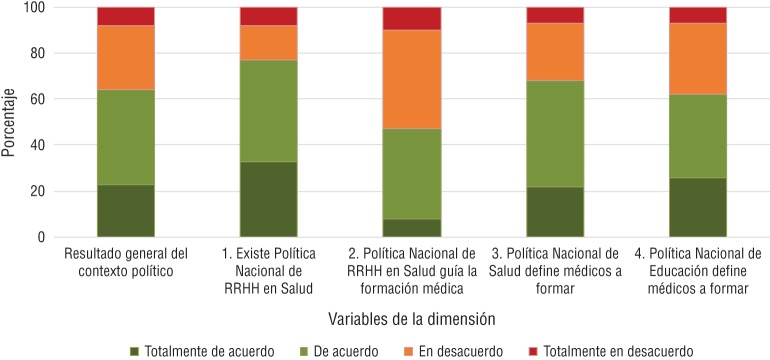
Porcentaje de respuestas a las variables de la dimensión contexto político de la educación médica, América Latina

Cuarta variable. Definición en la política nacional de educación del tipo de médicos que se debe formar. El 62,2% opinó favorablemente; este porcentaje fue ligeramente menor que el de la política nacional de salud (68,0%). En la figura 1 aparece el resultado general de esta dimensión (64,0%) y de sus cuatro variables.

### Segunda dimensión. Contexto económico de la educación médica

Quinta variable. Suficiencia de fondos públicos para la educación médica. El 15,5% de las escuelas consideró que los fondos públicos que proporciona el Estado son suficientes, y de estas, sólo 19% estuvo totalmente de acuerdo. El 84,5% restante de las escuelas estuvo en desacuerdo, de las cuales 32% estuvo en total desacuerdo.

**FIGURA 2.**
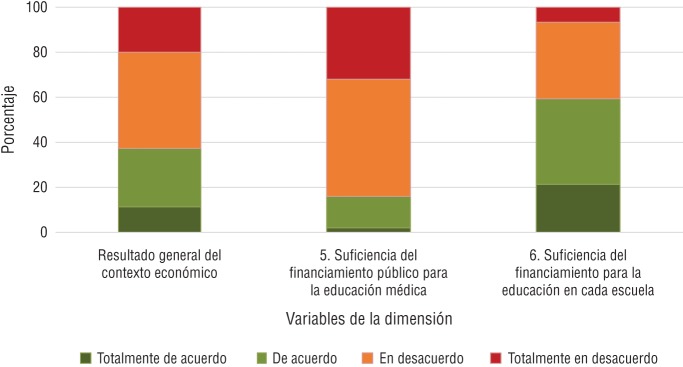
Porcentaje de respuestas a las variables de la dimensión contexto económico de la educación médica, América Latina

Sexta variable. Suficiencia de los recursos financieros que administra cada escuela. El 58,8% de las escuelas consideró que los recursos que administran son suficientes y 41,2% restante, insuficientes, pero hubo diferencias significativas entre escuelas públicas y privadas (32,2 y 82,3%, respectivamente, P < 0,01). En la figura 2 se presenta el resultado global de esta dimensión y de sus dos variables. En general, el contexto económico es favorable sólo para 37% de las escuelas.

### Tercera dimensión. Contexto regulatorio de la educación médica

Séptima variable. Existencia de coordinación entre los sectores de educación y salud para la planificación de médicos. El 41,3% de las escuelas refirió que hay mecanismos de coordinación entre ambos sectores, pero sólo 10,5% estuvo totalmente de acuerdo.

***Fuente:*** base de datos del estudio.

***Fuente:*** base de datos del estudio.

**FIGURA 3.**
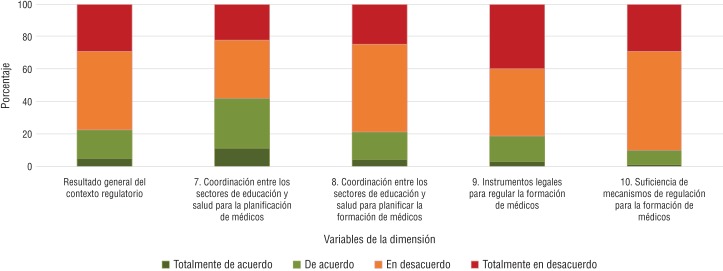
Porcentaje de respuestas a las variables de la dimensión contexto regulatorio, América Latina

***Fuente:*** Base de datos del estudio.

**FIGURA 4.**
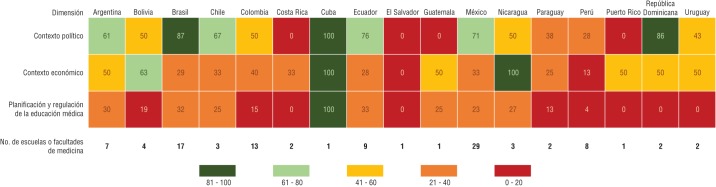
Porcentaje de respuestas favorables de las escuelas de medicina en las tres dimensiones estudiadas por país, América Latina

***Fuente:*** Base de datos del estudio.

Octava variable. Efectividad de la coordinación intersectorial para la planificación de médicos. Sólo 20,5% de las escuelas consideró efectiva la coordinación entre ambos sectores, y de estas, 3,9% estuvo totalmente de acuerdo y 16,6% solo de acuerdo.

Novena variable. Existencia de instrumentos legales para regular la formación de médicos. Sólo 18,6% de las escuelas indicó que existen instrumentos legales necesarios, como leyes, reglamentos y normas, para regular la formación de médicos y 81,4 estuvo en desacuerdo.

Decima variable. Suficiencia de mecanismos de regulación para la formación de médicos. Sólo 9,7% de las escuelas calificó de suficientes los mecanismos para regular el número de médicos que se forman para satisfacer las necesidades nacionales.

La figura 3 incluye el resultado global de esta dimensión y de sus cuatro variables. Sólo 23% de las escuelas consideró que existe suficiente regulación y planificación de la educación médica.

La figura 4 muestra sólo los resultados generales de las tres dimensiones por país. Se puede observar que la regulación de la educación médica es la más crítica de las tres, ya que, excepto en un país, en los 16 restantes 40% o menos de las escuelas opinaron favorablemente. También se aprecia que sólo en un país los resultados de las tres dimensiones fueron muy favorables y, por el contrario, en otro, las tres fueron muy desfavorables.

## DISCUSIÓN

Los OPS ha recomendado desde hace tiempo a los Estados miembros que definan políticas públicas para la formación de recursos humanos para la salud. Los resultados del presente estudio parecen reflejar un efecto favorable de esta exhortación, ya que 77% de las 105 escuelas participantes indicó que existen políticas públicas para formar recursos humanos de salud y 68%, que las políticas nacionales de salud orientan sobre el tipo de médicos que se necesita formar.

Sin embargo, el análisis de los resultados del contexto político por países aun muestra heterogeneidad, pues en seis países se perciben debilidades marcadas en la definición de políticas públicas para la formación de médicos, en cuatro están medianamente definidas, y en siete la definición es mejor. Si bien se trata de opiniones y no de otro tipo de evidencia, los resultados sugieren que todavía puede ser necesario que los organismos internacionales de cooperación, como la OPS, sigan prestando apoyo a los países para que definan políticas públicas efectivas que orienten la formación médica y tengan en cuenta las políticas de financiamiento y empleo, como regularmente lo hacen países pertenecientes a la OCDE, ya que también influyen en la contratación y distribución de médicos en los sistemas de salud (16,17).

La situación encontrada en esta dimensión representa un espacio de oportunidades para que las escuelas de medicina colaboren activamente con los sectores de salud y educación en la formulación de políticas públicas que sirvan de marco para la educación que imparten.

Respecto al contexto económico, la gran mayoría de escuelas (84%) consideró que los fondos públicos que el Estado invierte en la educación médica son insuficientes. Sin embargo, al analizar la suficiencia de recursos financieros que administra cada escuela, los resultados indican que la situación es menos crítica, ya que 59% de las escuelas consideró que son suficientes, aunque se debe destacar que esta opinión fue significativamente menor en las escuelas públicas (35%) que en las privadas (82%). Estos resultados parecen confirmar la percepción de que las escuelas públicas de medicina afrontan una situación financiera frágil, que puede limitar la adquisición de materiales, equipo y tecnología, así como la contratación de personal docente, y poner con ello en riesgo la calidad de la educación que imparten.

Los estudios sobre economía de la educación señalan que los fondos públicos destinados a ella son gastos de inversión más que de consumo y que la tasa de retorno de dicha inversión es muy alta, para las personas que reciben la educación y para la sociedad que la financia (18). Estos resultados sugieren que es necesario abogar por una mayor inversión pública en formación de médicos, tanto para garantizar la calidad de su enseñanza, como para formarlos en número suficiente.

La educación médica tiene un alto costo y a escala mundial se estima que el costo medio de formar a un médico general asciende a USD 100 000 (19). Sin embargo, en América Latina sólo algunos países, como Colombia, han estimado que los costos para formar un médico en escuelas públicas se sitúa alrededor de los USD 50 000 dólares y que en las escuelas privadas suele ser mayor (20). La falta de información sobre estos costos limita la gestión de fondos públicos para la educación médica, lo cual se debe subsanar investigando.

Llama la atención que los resultados generales del contexto político (64%) y del contexto económico (37%) muestren grandes diferencias, lo que puede traducir la asimetría existente entre la capacidad para formular políticas públicas de recursos humanos para la salud y la determinación política de asignar financiamiento público para ejecutarlas.

En cuanto a la regulación de la educación médica se encontró una situación crítica, ya que sólo 23% de las escuelas consideró que existe coordinación intersectorial entre salud y educación así como mecanismos e instrumentos legales para impartirla. La planificación de personal de salud parece encontrase en mejor situación que la regulación, ya que 42% de las escuelas opinó que hay coordinación entre los sectores de educación y salud para la planificación nacional de médicos y 21%, que la coordinación es efectiva y, en cambio, solo 19% indicó que existen instrumentos legales para la regulación y 10%, que dichos instrumentos son suficientes.

La regulación y la planificación son claves para formar a la cantidad de médicos generales que necesitan los sistemas de salud y estos resultados sugieren que aún queda un largo camino por recorrer para regular y planificar la educación médica en varios países de América Latina. Esta situación debe ser afrontada conjuntamente por instituciones formadoras y empleadoras para mejorar la disponibilidad y distribución de médicos generales a largo plazo y reducir sus carencias en la atención primaria y en algunas especialidades (21,22).

Los resultados de este estudio constituyen una aproximación al contexto político y económico de la educación médica de pregrado, así como a sus mecanismos de regulación en algunos países de la Región de las Américas. Las tres dimensiones estudiadas se relacionan con factores extrínsecos que pertenecen al ámbito de la responsabilidad estatal e influyen en la tarea educativa que realizan las escuelas de medicina y en el cumplimiento de su misión social (23).

Como las escuelas no se seleccionaron por muestreo aleatorio y el número de participantes fue bajo, los resultados de ese estudio no pueden ser extrapolados y generalizados y han de interpretarse con cautela. No obstante, describen un panorama general de las dimensiones estudiadas y plantean nuevas interrogantes que pueden abordarse con otras investigaciones. Asimismo, ayudan a identificar áreas de oportunidad tanto en el terreno técnico, como en el de la política pública, para mejorar la formación de médicos generales en América Latina.

## Agradecimientos.

Los autores agradecen a todos los Decanos de las facultades y escuelas de medicina que aceptaron formar parte de este estudio, sin cuya colaboración no hubiera sido posible.

## Financiación.

Este estudio no requirió de ningún tipo de financiamiento.

## Contribuciones de los autores.

Todos los autores participaron en el diseño del estudio original, la recopilación, análisis e interpretación de datos, así como en la redacción, revisión y corrección del manuscrito final para su aprobación.

## Declaración.

Las opiniones expresadas en este manuscrito son responsabilidad del autor y no reflejan necesariamente los criterios ni la política de la RPSP/PAJPH y/o de la OPS.
